# The Electronic Community Park Audit Tool (eCPAT): Exploring the Use of Mobile Technology for Youth Empowerment and Advocacy for Healthy Community Policy, Systems, and Environmental Change

**DOI:** 10.3389/fpubh.2018.00332

**Published:** 2018-11-20

**Authors:** Gina M. Besenyi, Benjamin Schooley, Gabrielle M. Turner-McGrievy, Sara Wilcox, Sonja A. Wilhelm Stanis, Andrew T. Kaczynski

**Affiliations:** ^1^Department of Kinesiology, College of Human Ecology, Kansas State University, Manhattan, KS, United States; ^2^Integrated Information Technology, College of Engineering and Computing, University of South Carolina, Columbia, SC, United States; ^3^Health Promotion, Education, and Behavior, Arnold School of Public Health, University of South Carolina, Columbia, SC, United States; ^4^Prevention Research Center, Arnold School of Public Health, University of South Carolina, Columbia, SC, United States; ^5^Exercise Science, Arnold School of Public Health, University of South Carolina, Columbia, SC, United States; ^6^Parks, Recreation, and Tourism, School of Natural Resources, University of Missouri, Columbia, MO, United States

**Keywords:** mobile technology, youth, participatory, empowerment, advocacy, usability, parks

## Abstract

Empowering and engaging youth in advocacy and participatory action research (PAR) for healthy community environments is an emerging approach to reducing the childhood obesity epidemic. Technology is a promising strategy for engaging youth in such efforts. The Community Park Audit Tool (CPAT) is user-friendly tool for evaluating the ability of parks to promote youth physical activity. Recently an electronic version of the tool (eCPAT) was developed and validated. The purpose of this study was to explore the use of eCPAT mobile technology on youth empowerment and advocacy. This study examined tool usability, youths' technology access, use, and readiness for PAR efforts, effectiveness of mobile technology on youth empowerment and advocacy, interaction effects between tool format and regular technology use, and tool format preferences. Youth ages 11–18 years were recruited and randomized into one of three study conditions: Control (no audit), paper (CPAT), and mobile technology (eCPAT). Intervention youth completed two park audits using assigned format. A subsample of youth in the Control group completed both CPAT and eCPAT audits for comparison. Independent samples *t*-tests and MANCOVAs explored differences in post-project levels of tool usability and empowerment and advocacy scores between groups. Multivariate linear regression analysis explored the interaction between Control, Paper, or eCPAT group membership and mean technology use in predicting empowerment and advocacy. Youth (*n* = 124) completed pre and post surveys. The majority of youth had access to technology (smartphone 77.4%, tablet/iPad 67.7%). Youth used mobile technology at least once a day to use apps (*M* = 7.8, *SD* = 3.2), browse the web (*M* = 6.3, *SD* = 3.3), and search for information (*M* = 6.3, *SD* = 3.5). Youth were also ready and willing to use technology for PAR (*M* = 3.42–3.59). No main or interaction effects were found for post-project levels of youth empowerment or advocacy. However, the eCPAT tool had high usability scores, was better liked, and was preferred by youth over paper-pencil methods. Mobile technologies are ubiquitous and a preferred strategy among youth for engagement in community change. Future studies should explore mobile technology as a potential strategy for engaging youth in ongoing PAR efforts to achieve successful engagement and advocacy in community healthy environmental change.

## Introduction

Over the past three decades, childhood obesity has emerged as a substantial public health issue given its association with an increased risk of a variety of health concerns, such as high blood pressure, high cholesterol, heart disease, diabetes, depression, and premature mortality (1, 2). Indeed national surveys indicate that childhood obesity rates have doubled in children and quadrupled in adolescents over the past three decades (3). In 2015–2016, 18.5% of American youth ages 2–19 years were obese, with obesity rates highest (20.6%) in 12 to 19 years old (4). Obesity is especially prominent in South Carolina where approximately 28% of children 2–5 years old and almost 1 in 3 high school students are overweight or obese (5, 6). This is particularly disconcerting because children who are overweight are 70% more likely to be overweight or obese as adults (7). Being physically active can significantly reduce the risk of childhood obesity and obesity-related chronic diseases (8, 9). However, youth physical activity participation declines with age (10, 11) with only 27% of U.S. students in grades 9–12 achieving recommended levels in 2015 (12).

Developing neighborhood and community policy, systems, or environmental (PSE) improvements that support physical activity, including the creation or enhancement of parks and recreation resources, is a promising solution to the childhood obesity crisis (13, 14). However, creating healthy community PSE change requires a transdisciplinary approach, involving participation from multiple parties including community members (15). Youth, in particular, should be recognized as competent citizens and community builders that can contribute to healthy community PSE change efforts, especially ones that directly affect them, by drawing upon their perspectives and improving municipal decision processes (16, 17). For example, in one prominent study, youth engaged in several activities to advocate for tobacco-free schools (e.g., testifying at board meetings, petitioning other youth) and of the seven schools that passed such policies, five had substantial evidence of youth involvement or initiation (17). Within this study, “adults readily acknowledged both the importance of having youth support and the leadership roles youth played in gaining support for the policy” [Ribisl et al.(17) p. 609–10]. Additionally, engaging and empowering youth in healthy PSE change efforts contributes to positive youth development and prepares them for roles as active citizens and future public health leaders (16–18). For example, Checkoway et al. described how members of the San Francisco Youth Commission have an increasing amount of influence in public policy at the municipal level and these efforts contribute to the youth's political and social development (16). They also stated that the youth “gain substantial knowledge of the community, practical skills in political advocacy and community organizing, and civic competencies for civil society” [Checkoway et al. (16) p. 1,159].

Participatory action research (PAR) is a common approach among social science and public health researchers that emphasizes community participation through collective inquiry, data collection, and action to address community-based issues (19, 20). Recent youth PAR models emphasize the need to promote positive youth development via youth empowerment through increased youth engagement in socioeconomic, public, and political community processes so that youth may be seen as valued community resources (21). Checkoway and colleagues agreed, stating that youth PAR is valuable because it can develop youth knowledge and perspectives on sociopolitical issues, encourage youth to exercise political rights, give a voice to an under-represented group, prepare youth for active democratic participation, and increase youth's ability to create community change (22). Indeed, several researchers suggest that youth PAR should be viewed as part of the social research movement focusing on community-based action for health (22, 23).

Past research indicates several common characteristics among youth PAR frameworks for successful community health promotion, including concepts of youth engagement, participation, and, most importantly, empowerment (19). Recognition of youth as vital assets that can foster socio-political change within the community is essential. This characteristic of youth PAR emphasizes the need for adults to accept youth as community change agents and provide a supportive environment that *engages* and challenges youth to take leadership roles. Also key is the understanding that as part of the empowerment process, youth must achieve *critical awareness* of community issues through some sort of knowledge or education component. Often, this requires the collection of information to better understand community needs and socio-political goals. Finally, the inclusion of youth in *meaningful participation* in action-oriented projects is critical. This step highlights the transfer of power from adults to youth to give youth a greater level of control as an important component to increasing youth empowerment.

A growing body of literature suggests that the use of innovative technology within a participatory action research (PAR) framework is a promising method to engage and empower youth participants in building healthy communities (24–30). For example, the Youth Empowerment Strategies (YES) Project focused on the use of Photovoice as a way to engage youth in social change efforts by capturing photos of strengths and issues within their environments (31). Their work with 122 youth ages 9–12 years old within 13 afterschool groups successfully fostered both individual and group-level empowerment through social action projects aimed at improving neighborhood conditions. Similarly, the Youth Neighborhood Mapping Initiative involved youth mapping neighborhood assets and liabilities and voicing their perspectives through the use of geographic information systems (GIS), photography, internet blogs, and other digital medias (32). The use of technology facilitated the youths' ability to express their perspectives, thereby engaging them in efforts to increase knowledge of community issues, raise community awareness, and advocate to affect change within their communities. Another study of 57 youth and five community partners through seven projects developed a conceptual model (e-PAR) for using technology within PAR to engage youth in community health promotion (26). These projects engaged youth with a variety of digital media (e.g., photography, videos, music, websites) to increase self-expression, communication, and skill building to improve youth empowerment, address community health issues, and create positive change.

Leveraging technology as part of youth PAR can facilitate diverse dimensions of youth empowerment (e.g., create a welcoming and safe environment, generate equitable power sharing, encourage participation in sociopolitical processes to effect change) by helping us to better understand how youth interact with their environment, (33) offer new ways and formats for youth to engage civically, (34) and provide youth with a vehicle for meaningful participation in the community (24, 35). A summary of benefits of utilizing technology within youth PAR frameworks is shown in Table 1. For example, technology has been shown to increase youth self-efficacy [overall (36) and explicitly for health-related PAR (24)], improve youth motivation for PAR, (34) increase youth voice in the community (assertiveness), (24) and provide political or social agency (34, 37). Technology can also improve youth empowerment by combating common issues with PAR. For example, Amsden and VanWynberghe (41) note that youth typically fail to understand what PAR really is. However, use of technology within youth PAR efforts can fight apathy (34), support reflective thought (38), make them more self-sufficient researchers (39), and increase youth civic engagement (24, 42). Additionally, youth PAR is often fraught with issues of lack of trust and power sharing between adults and youth (43), yet technology can improve relationships with adults through increased efficacy (24), reduced youth anxiety (24), improved communication (40), and the promotion of equitable power sharing through increased youth control (24, 35).

**Table 1:** Summary of benefits of technology within youth PAR frameworks.

Increases self-efficacy Fights apathy/improves motivation Facilitates youth self-expression Provides meaningful participation Increases youth voice within the community Improves youth-adult communication Promotes equitable power sharing (increased youth control) Provides political or social agency Improves access to resources Improves research capabilities Increases civic engagement

*References (19, 20, 24, 26, 32, 34–40)*.

While promising, youth advocacy for healthy community PSE change is an understudied and under-evaluated approach (44). Further, a gap remains between the development of youth-oriented technology tools and the inclusion of such tools within youth PAR frameworks (27). The process of improving communities to promote physical activity and health will take time, but developing adequate technology tools and preparing today's youth to be the future leaders of healthy communities is a crucial first step (17, 27). The present study builds on two previous projects: the development of the Community Park Audit Tool (45) (CPAT) and the Healthy Young People Empowerment (HYPE) Project (46). The CPAT project engaged 34 community stakeholders from diverse backgrounds (parks and recreation, health care, planning, education, private business, parents, teenagers, etc.) in a year-long study to develop and test a park audit tool to assess the potential of parks to promote physical activity (45). The project involved three workshops and testing of the CPAT in 66 parks across Kansas City, MO. The resulting tool was six pages long, included four sections (park information, access, and surrounding neighborhood, park activity areas, and park quality), and demonstrated strong inter-rater reliability by community stakeholders (45). As described by participants, this process also resulted in a variety of important secondary outcomes related to community building, awareness, advocacy, and substantially improved perceptions of the importance of parks for community health (45).

The HYPE Project was developed to enhance the capacity of adolescents (12–17 years, especially from low income and minority backgrounds) to plan and implement PSE change projects centered around community healthy eating and active living needs (46). HYPE was guided by the MATCH model of health promotion as well as empowerment and positive youth development theories within a social ecological framework (23, 47, 48). The HYPE Project consists of facilitator-led, 60 min sessions through five progressive stages (Think, Learn, Act, Share, Evaluate) and culminates in a youth-led community PSE change project (46). As of today, the HYPE Project has been implemented with 258 youth within 21 youth groups across 15 counties in South Carolina. Of these, several groups have utilized the CPAT tool as part of their action planning. Preliminary results of the HYPE Project indicate youth saw increases in community awareness, empowerment for, and engagement in youth-led action planning for healthy eating and active living (46). As well, youth qualitative feedback indicated the CPAT was helpful in collecting and using important environmental data in their PSE change efforts. However, youth participants felt that mobile technology would be an easier and considerably more engaging format to collect park data than the current paper-and-pencil method (46). Therefore, to further advance this research and practice agenda, developing and testing the viability of an electronic version of the community park audit tool (eCPAT) among youth is an important next step.

The purpose of this study was to explore the use of eCPAT mobile technology on youth empowerment and advocacy for healthy community policy, systems, and environmental change efforts. Our goal was to understand differences between youth using mobile technology or paper-pencil tools within a PAR framework. Specifically, we explored four research questions:

1. Which tool format (mobile technology vs. paper-pencil) has higher levels of usability?
2. What is the effectiveness of using mobile technology (vs. paper-pencil or no treatment) on indicators of youth empowerment or advocacy?
3. Does regular technology use interact with tool format to predict levels of youth empowerment or advocacy?
4. What are youth tool impressions and preferences?

## Methods

### Conceptual model

**Figure 1:**
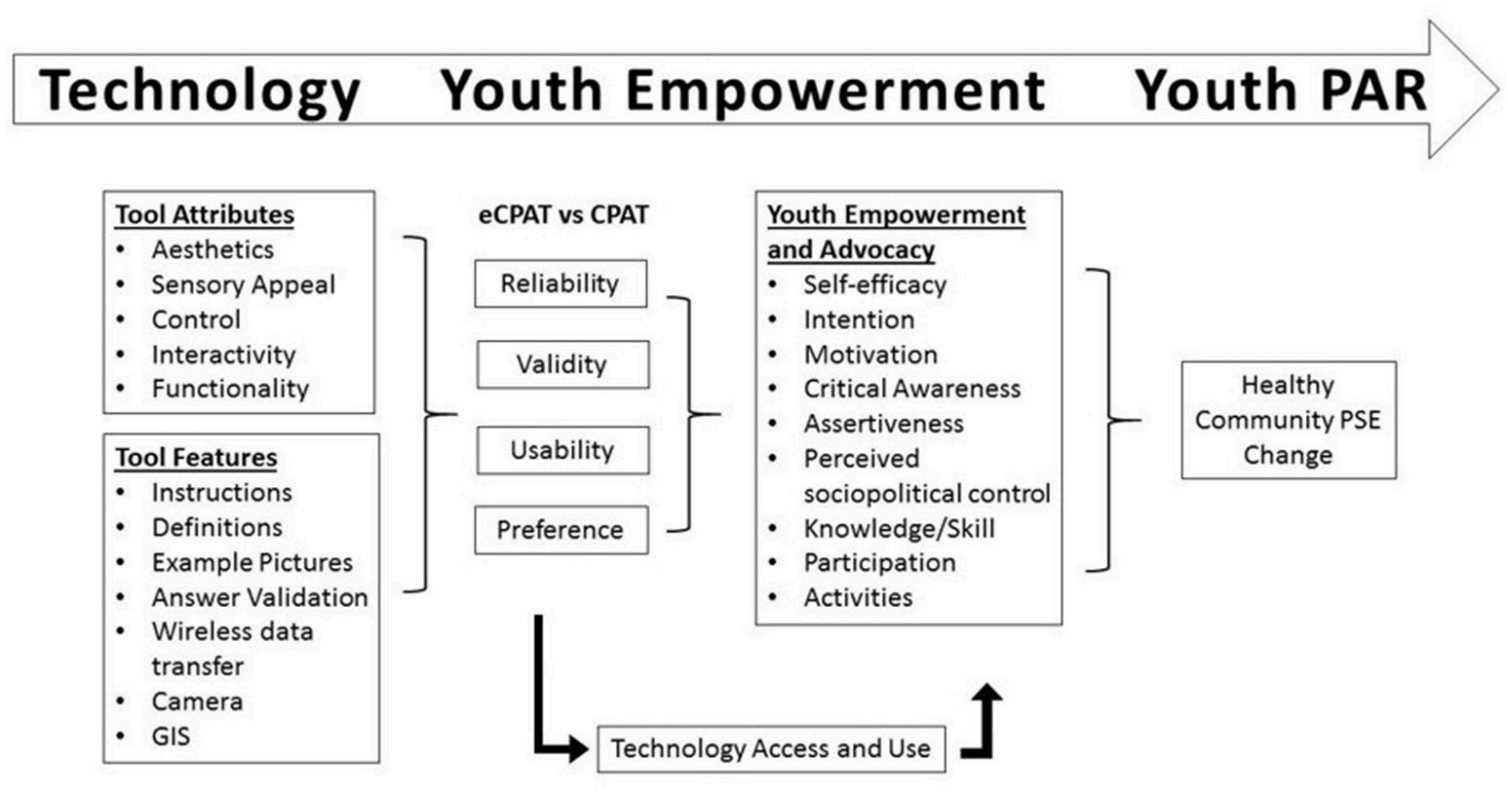
Technology, youth empowerment, and participatory action research conceptual model.

This study was guided by technology user engagement and youth empowerment theories (discussed further below) (23, 26, 49, 50). The conceptual model for this study illustrated in Figure 1 depicts the process of developing and testing mobile application technology to improve indicators of youth empowerment for healthy PSE change efforts (e.g., self-efficacy, motivation, critical awareness, perceived sociopolitical control). As shown in the left side of the model, development of the eCPAT mobile application was accomplished by incorporating key attributes of technology that influence user engagement (or disengagement) such as interface aesthetics, sensory appeal, control, and interactivity, as well as improvement of functionality through application features such as instructions, definitions, examples, and photo capabilities (49). Interface attributes and application features, along with previously validated CPAT content, (45). provided the foundation to create a highly usable eCPAT application for use by youth. Development of the eCPAT app is discussed in greater detail below.

According to the model (Figure 1), it was expected that through use of the eCPAT mobile application, youth will experience enhanced technology benefits for participating in PAR efforts (24, 34, 35). Technology benefits are expected to lead to improvements in dimensions of youth empowerment and advocacy, such as increased youth self-efficacy and motivation for becoming involved in community-based efforts, increased youth knowledge and critical awareness of community issues, and heightened perceptions of sociopolitical control and assertiveness for making healthy community changes (26–28). As indicated in the model, some research has found that youth's access and use of technology can impact resulting levels of civic engagement (51). Likewise, in one study of adults, mobile technology use was shown to be a positive predictor of civic participation. However, this effect was moderated by mobile technology competence in that those who felt more competent with technology showed stronger positive relationships than those who had lower technology competence (52). Therefore, as part of the conceptual model, this study will explore the potential moderating effect that regular technology use might have on post-project levels of empowerment and advocacy. Finally, improvements in youth empowerment are expected to positively influence youth advocacy and participation in healthy community PSE change efforts in the future (23, 28, 50). While the conceptual model above represents the entire process from technology development to youth engagement with technology to actual participation in PSE change efforts, this study did not involve a full intervention that addressed all of these stages. Rather, this study represented key initial stages of the conceptual model including the development and testing of the innovative technology vital for successful youth empowerment as well as preliminary analyses of the effect of engaging in data collection with the eCPAT app.

### eCPAT app development

Multiple iterative stages were used to comprehensively develop an eCPAT app (53). Briefly, a systematic literature review of youth, technology, and health advocacy identified theoretical frameworks and key methodologies for developing mobile applications to engage youth in health promotion efforts (23, 26, 49, 50). To further inform application development, key informant interviews (*n* = 5) were conducted with experts in youth advocacy for obesity prevention, health information technology, and technology within parks and recreation settings about topics related to application format, design, functionality, and preferred operating systems and mobile devices. Linking this information to technical programming design, a team of health promotion and computer science academics used PhoneGap (a cross-platform framework that allows application design for both Android and iOS platforms) to create the eCPAT application for use on Android tablets. Technical application development phases followed standard system design protocol and included a system requirement analysis, software design, program coding, and unit alpha (capacity) testing by computer programmers. Concurrently, a Microsoft SQL database was designed to house wireless data transfer from the eCPAT app upon data submission. Upon application and server design completion, a second round of extensive capacity field-testing of both the eCPAT application and wireless data transfer and storage were conducted. Further details about the development and testing of the eCPAT app can be found elsewhere (53). A comparison between the CPAT and the newly developed eCPAT formats can be found in Table 2. Key improvements of the mobile technology format include enhanced interface attributes such as sensory appeal (e.g., touchscreen, colorful font/graphics), control (e.g., enhanced navigation), and interactivity (e.g., answer validation, messages). As well, the eCPAT app included additional technology functionality such as built-in instructions and examples, ability to take pictures, GPS/GIS data collection, wireless data transfer (eliminates the need for manual data entry), and acknowledgment of successful completion.

**Table 2:** Comparison of audit tool formats.

	**CPAT**	**eCPAT**
Format	Paper	Electronic
**Interface Attributes**	
Aesthetics	Black and white paper	Color with graphics
Sensory appeal	No	Touchscreen
Control	Limited	Yes
Interactivity	No	Yes
Functionality	Limited	Yes
**Features**	
Instructions	Limited within tool (Separate training manual)	Yes
Definitions	Limited within tool (Separate training manual)	Yes
Example pictures	None within tool (Separate training manual)	Yes
Camera	No	Yes
GIS	No	Yes
Answer validation	No	Yes
Wireless data transfer	No	Yes
Successful completion message	No	Yes

### Study setting

This study occurred in Greenville County, South Carolina. Greenville County is an important setting for this study due to significantly high rates of obesity. The state of South Carolina is ranked 42nd in the nation for obesity, with 30.8% of the population having a BMI of 30 or greater. Among youth in South Carolina, almost 1 in 3 high school students is overweight or obese (54). Likewise, in South Carolina, almost 60% of high school students and almost 50% of middle school students are not physically active at least 60 min/day on 5 or more days/week (54). These problems are especially prominent in Greenville County, where 41% of students are overweight (19%) or obese (22%) (55). Additionally, Greenville County was determined as an ideal location for this study given that it leveraged the study team's prior partnerships with parks and youth agencies and extended previous research efforts with the Greenville County community.

### Study design and participants

**Figure 2:**
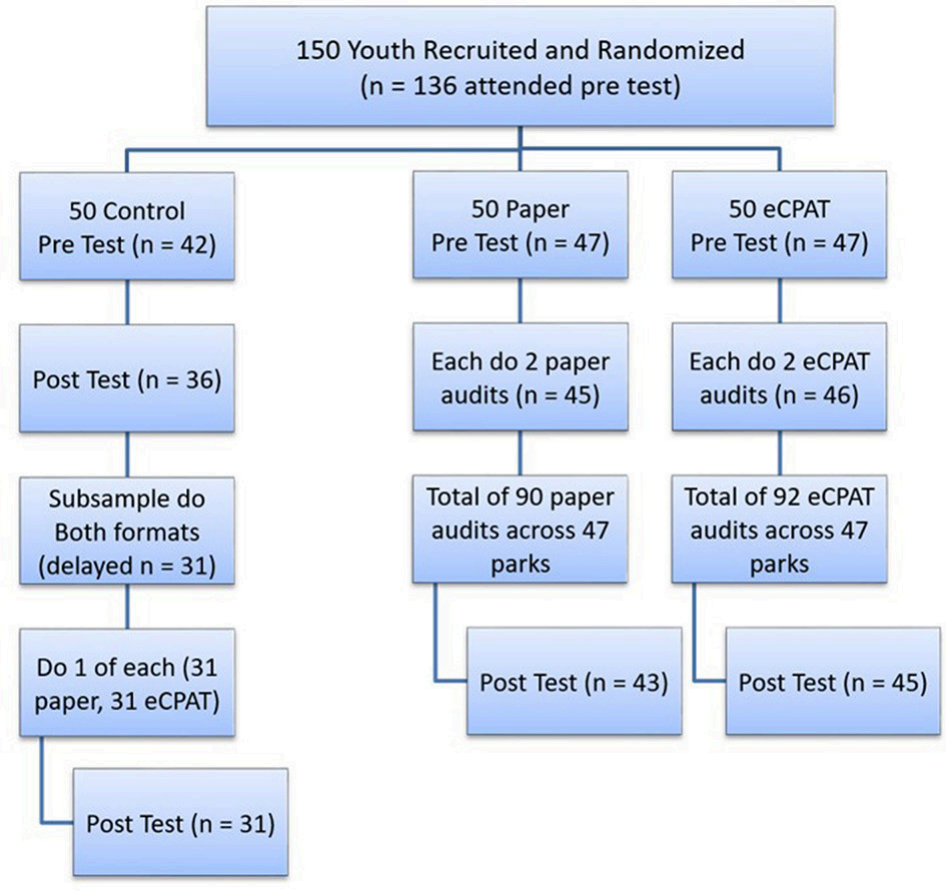
Study design and randomization.

This study utilized a randomized untreated delayed control group design with pre-test/posttest as shown in Figure 2. With the assistance of Greenville County Parks, Recreation, and Tourism, the City of Greenville Parks and Recreation Department, and LiveWell Greenville, 150 youth 11–18 years of age were recruited through a variety of methods to garner a broad cross-section of participants. Recruitment methods included distribution of a recruitment flier through email and hard copies to Greenville County schools, after school groups, and parks and recreation programs, as well as a recruitment booth at the opening ceremony of the Park Hop summer program. All recruitment materials (emails, flyers, QR code) directed parents and youth to an event-planning website (EventBrite) for project registration. The website included an overview and specific aims of the project, youth project requirements and incentives, anticipated project data collection dates, and a link to a website with a full project description. The study was open to youth of all racial and ethnic groups and inclusion criteria encompassed those 11–18 years old, living in Greenville County or attending a Greenville County school, and being able to hear, speak, and comprehend English. Blocked randomization using a random number generator was used to allocate the 150 youth into one of three study conditions (i.e., Paper, eCPAT and Control, as described further below) ensuring similar group sizes (approximately 50 per group). However, to help reduce contamination between conditions, youth within the same family were assigned to the same condition. This study was carried out in accordance with the recommendations of the Office of Research Compliance, University of South Carolina. This protocol was approved by the University of South Carolina Institutional Review Board. All parents gave written informed consent and youth written informed assent in accordance with the Declaration of Helsinki.

### Data collection

Data for this study were collected in June 2014. Pre and post data collection numbers are shown in Figure 2. Prior to project participation, all youth were given a pre-test survey that gathered baseline information about youth empowerment and advocacy indicators, technology access and use, and demographics. Youth in the Paper and eCPAT conditions were considered part of the “intervention,” which included an hour-long, condition-specific project meeting followed by independent youth collection of observational data within parks using either paper or mobile technology formats. The project meeting included an overview of the project (15 min) and audit tool training for their assigned tool (15 min) that consisted of basic instructions, definitions, and information about answering questions. Youth also completed an on-site practice park audit (30 min) with their assigned tool at a park adjacent to the community center.

Observational park audits took place in 47 parks in Greenville County, SC. Project parks were selected to represent a diverse mix of quality, size, features, and geographic dispersion while staying within a 30-mile radius from the City of Greenville center to alleviate travel concerns. Youth in the Paper and eCPAT groups were randomly assigned the name of two parks and asked to independently complete a park audit at each site using their assigned audit format (Figure 2). To alleviate safety concerns, all park audits were completed at assigned times under the supervision of research staff. Youth in the eCPAT app group were provided Google Nexus 10 tablets onsite, while youth in the Paper condition were provided with pencils, clipboards, and paper copies of the CPAT tool. After completion of their assigned park audits, youth in the Paper and eCPAT conditions completed a posttest survey specific to their experimental condition.

Youth in the Control group received no treatment during the main portion of the study and were also given a posttest. Approximately 1 week after completion of the project posttest, a subsample of youth (*n* = 31 from the Control group were recruited to participate in a “Both” group (Figure 2). Similar to the Paper and eCPAT conditions, youth in the Both group completed a brief project meeting where they received training and audit tool practice, with the exception that this condition utilized both paper and mobile technology formats. Youth in the Both group were then assigned two park names and asked to complete one park audit using the eCPAT and one using the paper-pencil CPAT. After completing the assigned park audits, youth in the Both group completed a project posttest. Youth received a $50 gift card for attending the initial project meeting, submitting their assigned park audits, and completing brief pre- and post-project surveys.

### Measures

All youth completed identical pre-project surveys and then condition-specific post surveys which included measures that captured constructs related to tool usability, impressions and preferences, technology readiness and use, as well as indicators of youth empowerment and advocacy. Usability of each tool (Paper or eCPAT) was captured in the post-project survey with a modified version of the System Usability Scale (SUS)(56) that was comprised of 10 items on a 5-point Likert scale (e.g., I thought the [tool] was easy to use; 1 = Strongly disagree, 5 = Strongly agree). SUS scores were computed according to standard protocols that resulted in values ranging from 0 to 100, with scores of 68 or higher signifying above average or usability, scores of 50–68 indicating marginal usability, and those below 50 indicating unacceptable usability (57–60). Overall impressions of audit tools were captured with a single item on a 5-point Likert scale (1 = Very negative, 5 = Very positive). Audit tool preferences were captured with a series of questions asking which tool they found easiest, most enjoyable, would want to use in the future, and liked the best.

Youth empowerment was captured within the pre- and posttest using the Individual Community-Related Empowerment (ICRE) scale shown to have high content validity (Lawshe's formula, CVR = 0.98) and internal consistency (α = 0.86) (50). The scale consisted of five dimensions that measured self-efficacy for making changes in the community (7 items, α = 0.88), intention to get involved in the community (4 items, α = 0.83), motivation to get involved in the community (3 items, α = 0.69), participation in community activities (3 items, α = 0.81), and critical awareness of issues in the community (1 item). This scale was assessed using a 5-point Likert scale (1 = Strongly disagree, 5 = Strongly agree) and included items such as “I have the knowledge and skills to influence my community” and “I am willing to get involved in my community.” Additionally, youth advocacy was captured using items from the evaluation of the Youth Engagement and Action for Health (e-Yeah) Program which were found to have moderate to good internal consistency reliability (61). The four dimensions related to youth advocacy for obesity prevention and included assertiveness for being a leader in the community (3 items, ICCs = 0.474, 0.524, 0.678), perceived sociopolitical control for making changes in the community (4 items, ICCs = 0.311, 1.0), history of advocacy activity (2 items, ICC = 0.154), and knowledge of resources (1 item). This scale was assessed on a 5-point Likert scale (1 = Strongly disagree, 5 = Strongly agree) and included items such as “I can talk with adults about issues I believe in” and “I enjoy participation because I want to have as much say as possible in my school or community.” A score for each youth empowerment or youth advocacy dimension was created by averaging items within each subscale.

Information about youth access to technology was captured with a single question asking youth to check the types of mobile technology they could access (e.g., smartphone, tablet, etc.). Technology use and readiness dimensions were captured with a modified version of the Media and Technology Usage and Attitudes Scale (MTUAS) (62). This scale assessed information related to regular technology use on a 10-point Likert scale (1 = Never, 10 = All the time) and included subscales that measured smartphone usage (9 items, α = 0.93), text messaging (3 items, α = 0.84), phone calling (2 items, α = 0.71), internet searching (4 items, α = 0.91), media sharing (4 items, α = 0.84), and video gaming (2 items, α = 0.83) (62). A composite technology use score was created by calculating a mean for each subscale (1-10) and then averaging the seven subscales. Mean technology use was categorized as high (>5) or low ( ≤ 5), designating differences in regular use between “several times per week” and “once per day.” In addition, four survey items were specifically created within the context of this project to better understand youth readiness/willingness to use mobile technology for healthy community PAR. The items were measured on a 5-point Likert scale (1 = Strongly disagree, 5 = Strongly agree) and asked specifically about whether the youth would use mobile technology to access community news, communicate with community leaders, voice opinions about changes, and advocate for community changes. Finally, youth demographic information was collected, including gender, date of birth, height, weight, race, ethnicity, and whether or not the youth received free or reduced lunch at school (a common proxy measure for low-income students).

### Analyses

To examine differences in tool usability, an independent samples *t*-test was used to test differences in mean usability scores between Paper and eCPAT conditions. To examine differences in post-project levels of youth empowerment and advocacy, factorial multivariate analyses of covariance (MANCOVAs) compared the mean posttest empowerment and advocacy dimension scores across the Control, Paper, and eCPAT conditions controlling for respective baseline levels of each construct. Separate models were conducted for youth empowerment (5 variables) and youth advocacy (4 variables) scales. Skewness and kurtosis values as well as box plots were obtained to examine the distributions of youth empowerment and youth advocacy variables. Outliers as identified by SPSS (i.e., interquartile range multiplied by 1.5) were removed prior to analyses (63). To understand potential moderating effects of regular technology use and readiness on the relationship between group condition and post-project levels of youth empowerment and advocacy, multivariate linear regression analyses explored the interaction between Control, Paper, or eCPAT group membership and mean technology use and readiness. Finally, descriptive statistics, including frequencies and percentages, explored youth impressions and preferences for the Paper or eCPAT tools among youth in the Both group that utilized both audit versions. All analyses were performed in SPSS 22 (Armonk, NY). Little evidence exists that would suggest the level of expected change from an intervention such as this, but the sample size of 50 youth per condition allows for detection of a moderate (0.60) effect size (at alpha = 0.05 and power = 0.80), which is a reasonable expectation for this pilot study (64).

### Results

A total of 136 youth participated in the study; however, 12 youth were lost to attrition resulting in a final sample of 124 youth. Youth participant characteristics by study condition are shown in Table 3. Youth ranged from 11 to 18 years of age (*M* = 13.6, *SD* = 1.7), with just over half (50.8%) of participants in middle school. Youth participants were fairly representative of the Greenville County population with respect to gender, race/ethnicity, and socioeconomic indicators (65). The majority of youth participants were female (62.1%), white (62.1%), and owned a bike (83.9%). Chi square and ANOVA tests for distribution of youth characteristics between study conditions indicated no significant differences between groups for gender [$\chi_{\left(2\right)}^{2}$ = 0.44, *p* = 0.80], age [*F*_(2, 133)_ = 0.79, *p* = 0.46], race [χ^2^_(8)_ = 4.96, *p* = 0.76], or free/reduced school lunch [$\chi_{\left(6\right)}^{2}$ = 9.70, *p* = 0.14].

**Table 3:** Youth participant characteristics.

**Characteristic**	**Total *n* (%)**	**Control *n* (%)**	**Paper *n* (%)**	**eCPAT *n* (%)**
Total	124 (100)	36 (29.0)	43 (34.7)	45 (36.3)
**AGE**				
Middle school (11–13 yrs)	63 (50.8)	19 (52.8)	22 (51.2)	22 (48.9)
High school (14–18 yrs)	61 (49.2)	17 (47.2)	21 (48.8)	23 (51.1)
**GENDER**				
Male	47 (37.9)	13 (36.3)	18 (41.9)	16 (35.6)
Female	77 (62.1)	23 (63.9)	25 (58.1)	29 (64.4)
**RACE**				
White	77 (62.1)	19 (52.8)	29 (67.4)	29 (64.4)
Black	31 (25.0)	11 (30.6)	9 (20.9)	11 (24.4)
Other	3 (2.4)	1 (2.8)	1 (2.3)	1 (2.2)
2 or more races	13 (10.5)	5 (13.9)	4 (9.3)	4 (8.9)
Hispanic/Latino	5 (4.0)	0 (0)	4 (9.3)	1 (2.2)
Free/reduced school lunch	23 (18.5)	8 (22.2)	10 (23.3)	5 (11.1)

**Figure 3:**
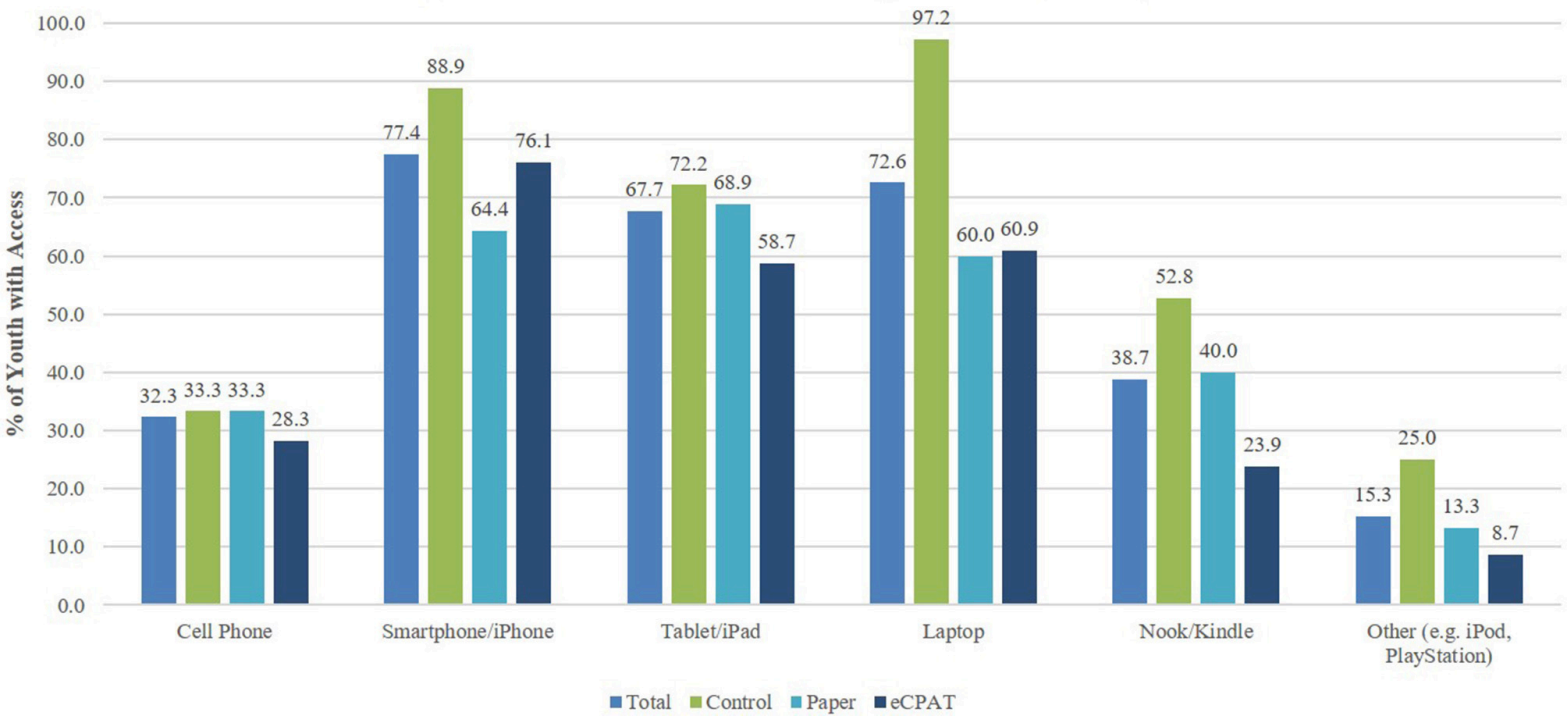
Youth mobile technology by study condition.

**Figure 4:**
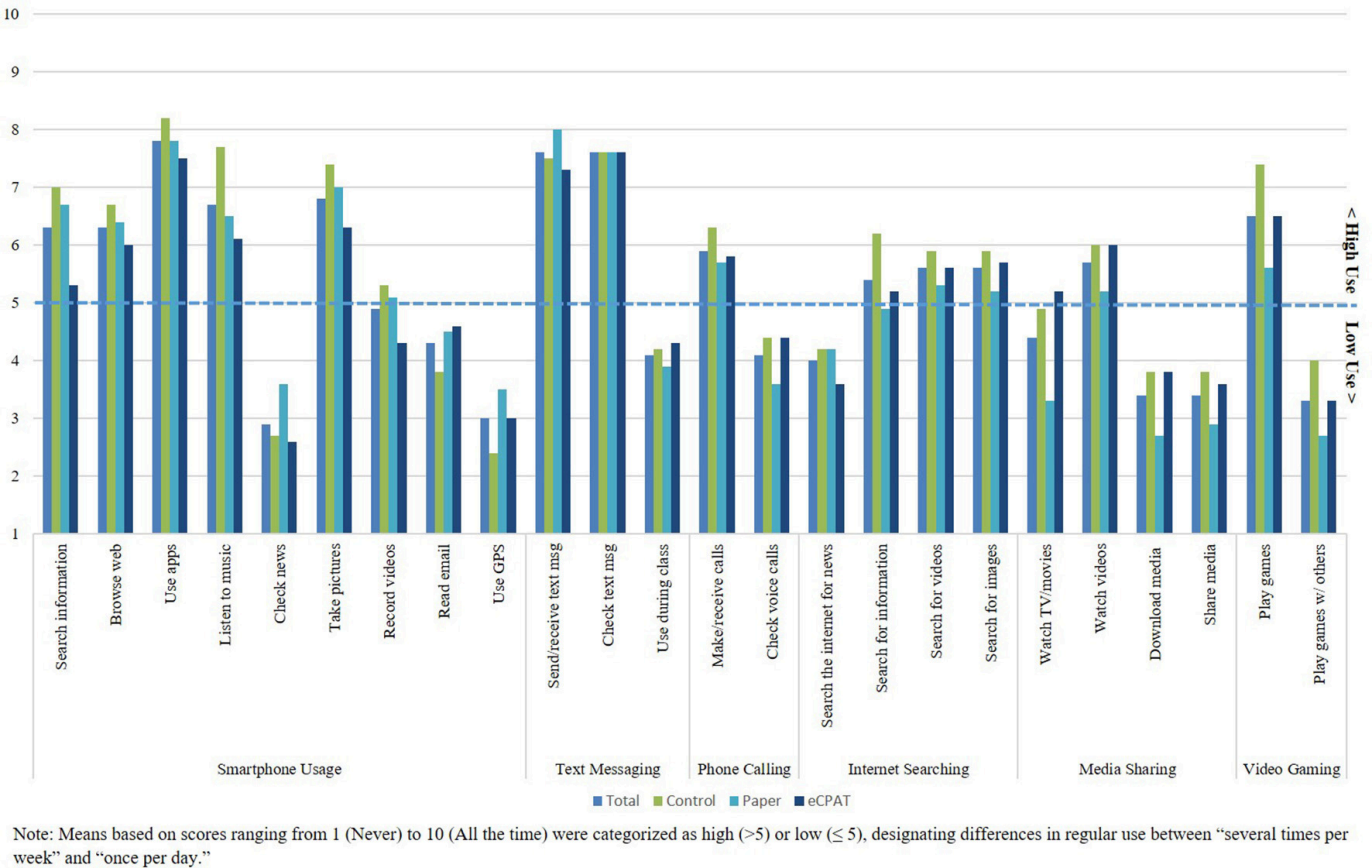
Mean mobile technology use by study condition.

As part of our study, we wanted to understand youth access to technology, regular technology use and readiness/willingness to use technology for community PAR activities. Results (shown in Figure 3) indicate that the majority of youth had access to a variety of mobile devices including a smartphone (77.4%), tablet or iPad (67.7%), and/or a laptop (72.6%). Average mobile technology use shown in Figure 4 indicates use of mobile technology by group on a scale of 1 (Never) to 10 (All the time). All youth indicated they regularly (>5; at least once a day) use apps (*M* = 7.8, *SD* = 3.2), check for text messages (*M* = 7.6, *SD* = 2.8), send/receive text messages (*M* = 7.6, *SD* = 2.7), take pictures (*M* = 6.8, *SD* = 3.1), listen to music (*M* = 6.7, *SD* = 3.6), play games (*M* = 6.5, *SD* = 3.0) browse the web (*M* = 6.3, *SD* = 3.3), and search for information (*M* = 6.3, *SD* = 3.5). Chi square and ANOVA tests for distribution of youth technology access and use between groups indicated no significant differences, with the exception of the Control group having slightly more access to laptops than the other groups [$\chi_{\left(2\right)}^{2}$ = 7.43, *p* < 0.05]. Overall, youth responded positively for being ready and/or willing to use technology for community PAR activities (Table 4). On average, youth tended to agree that that they would use a mobile device to find out what's going on in their community (*M* = 3.4, *SD* = 1.0), to communicate with school or community leaders (*M* = 3.5, *SD* = 1.1), to voice their opinions about community changes (*M* = 3.5, *SD* = 1.1), and to convince people to make school or community changes (*M* = 3.6, *SD* = 1.1). One-way ANOVAs indicated no significant differences between groups regarding baseline technology readiness measures (*p*-values ranged from 0.07 to 0.54).

**Table 4:** Youth's readiness to use mobile technology for participatory action research.

**Characteristic**	**Total *n* = 124**	**Control *n* = 36**	**Paper *n* = 43**	**eCPAT *n* = 45**
Mobile technology readiness	M (*SD*)	M (*SD*)	M (*SD*)	M (*SD*)
I would use a mobile device to find out what's going on in my community	3.4 (1.0)	3.4 (1.3)	3.5 (0.5)	3.4 (1.0)
I would use an app on a mobile device to communicate with school or community leaders	3.5 (1.1)	3.4 (1.3)	3.6 (0.9)	3.3 (1.2)
I would use an app on a mobile device to voice my opinions about changes that should be made in my community	3.5 (1.1)	3.5 (1.3)	3.6 (0.9)	3.3 (1.0)
I would use an app on a mobile device to convince people to make changes in my school or community	3.6 (1.1)	3.6 (1.2)	3.9 (0.7)	3.4 (1.1)

*Scores represent means on a 5-point scale (1 = Strongly disagree, 5 = Strongly agree)*.

Our first research question explored differences in youth perceptions of tool usability between paper and mobile technology formats. Mean usability scores for both the Paper and eCPAT group were above 68 out of 100. According to usability scale scoring protocols this indicates that both tools had above average usability (57, 58) eCPAT usability scores averaged 77.1 (*SD* = 11.1) while Paper usability scores averaged 74.4 (*SD* = 15.0). However, an independent samples *t*-test indicated that this difference was not statistically significant (*t*_(85)_ = −0.995, *p* = 0.32).

Our second research question examined the effectiveness of using mobile technology tools for healthy community PAR on post-project levels of youth empowerment (i.e., self-efficacy, intention, participation, motivation, critical awareness) and advocacy (i.e., assertiveness, perceived sociopolitical change, advocacy activity, knowledge). Nine participants were identified as outliers for the youth empowerment analysis and 12 participants were identified as outliers for the youth advocacy analysis. *Post hoc* outlier comparison tests found no differences in age or gender between study groups. Pre and post means for youth empowerment and advocacy variables by study condition can be found in Table 5. Both pre and posttest youth answered positively (>3) for most indicators of youth empowerment or advocacy with the exception of participation in advocacy activity where youth were skewed toward disagreement (<3). Mean differences between pre- and posttest scores illustrate that youth in the Control condition saw positive changes in four of nine youth empowerment and advocacy variables. Youth in the Paper condition saw positive changes in seven out of the nine dependent variables. Youth in the eCPAT group saw positive changes in six out of nine empowerment and advocacy variables. Factorial multivariate analyses of covariance (MANCOVAs) controlling for baseline indicated no significant differences in post-project youth empowerment [Pillai's Trace V = 0.10, *F*_(10, 204)_ = 1.12, *p* = 0.35)] or youth advocacy [Pillai's Trace V = 0.08, *F*_(8, 202)_ = 1.09, *p* = 0.37)] variables between groups.

**Table 5:** Mean differences in youth empowerment and advocacy.

**Study condition**	**Empowerment or advocacy variable**	**N^a^^b^**	**Pre Mean**	**Std. deviation**	**Post mean**	**Std. deviation**	**Mean difference**
Control	Self-efficacy	33	3.97	0.52	3.86	0.48	−0.12
	Intention	33	4.14	0.79	3.98	0.67	−0.16
	Participation	33	3.62	0.87	3.65	0.80	0.02
	Motivation	33	4.16	0.78	4.16	0.66	0.00
	Critical awareness	32	3.21	0.98	3.27	1.04	0.07
	Assertiveness	32	4.03	0.59	4.01	0.57	−0.02
	Perceived sociopolitical control	32	3.82	0.64	3.64	0.60	−0.18
	Advocacy activity	32	2.02	0.80	2.06	0.70	0.05
	Knowledge	32	3.91	0.84	3.97	0.82	0.06
Paper	Self-efficacy	42	4.06	0.57	4.10	0.58	0.04
	Intention	42	4.15	0.76	4.08	0.73	−0.07
	Participation	42	3.79	0.88	3.92	0.91	0.13
	Motivation	42	4.27	0.67	4.17	0.56	−0.10
	Critical awareness	42	3.69	1.00	3.79	1.05	0.10
	Assertiveness	42	3.99	0.60	4.05	0.61	0.06
	Perceived sociopolitical control	42	3.64	0.64	3.70	0.62	0.07
	Advocacy activity	42	2.18	0.86	2.30	0.82	0.12
	Knowledge	42	3.95	0.85	4.10	0.76	0.14
eCPAT	Self-efficacy	40	3.76	0.56	3.83	0.48	0.07
	Intention	40	3.94	0.58	3.88	0.55	−0.05
	Participation	40	3.68	0.66	3.90	0.69	0.22
	Motivation	40	3.92	0.68	4.10	0.59	0.18
	Critical awareness	40	3.51	0.97	3.63	0.93	0.11
	Assertiveness	37	3.61	0.62	3.53	0.51	−0.08
	Perceived sociopolitical control	37	3.38	0.59	3.36	0.52	−0.01
	Advocacy activity	37	1.73	0.69	1.81	0.67	0.08
	Knowledge	37	3.35	0.92	3.59	0.72	0.24

a *9 outliers removed prior to empowerment analyses*.

b *12 outliers removed prior to advocacy analyses*.

In relation to the third research question, we wished to understand whether youth's regular use (or non-use) of technology moderated the effect that using mobile technology for community PAR had on youth empowerment or advocacy indicators. The mean technology use score for all youth in the study (*M* = 5.1, *SD* = 2.0), indicated that youth generally used mobile technology at least once per day. One-way ANOVA results indicated no significant differences in mean technology use across groups [*F*_(2, 119)_ = 1.27, *p* = 0.28]. Multiple linear regression explored the interaction between study conditions (Control, Paper, eCPAT) and regular technology use (high vs. low) on posttest levels of youth empowerment and advocacy. Descriptives for interaction models for youth empowerment are shown in Table 6 and advocacy in Table 7. No significant main effects for the interaction models were found for youth empowerment (Pillai's Trace = 0.15, *F*_(10, 194)_ = 1.605, *p* = 0.107) or youth advocacy variables (Pillai's Trace = 0.11, *F*_(8, 192)_ = 1.449, *p* = 0.179).

**Table 6:** Youth empowerment by study condition and technology use.

**Dependent variable youth empowerment**	**Study condition**	**Technology use**	**Mean**	**Std. error**	**95% Confidence interval**	
					**Lower bound**	**Upper bound**
Self-efficacy	Control	Low	3.665^a^	0.111	3.444	3.886
		High	3.921^a^	0.090	3.743	4.099
	Paper	Low	3.990^a^	0.084	3.824	4.156
		High	4.182^a^	0.098	3.988	4.376
	eCPAT	Low	4.046^a^	0.091	3.865	4.227
		High	3.847^a^	0.094	3.660	4.034
Intention	Control	Low	3.861^a^	0.148	3.567	4.156
		High	4.014^a^	0.119	3.777	4.251
	Paper	Low	3.943^a^	0.111	3.722	4.164
		High	4.173^a^	0.130	3.915	4.431
	eCPAT	Low	4.002^a^	0.121	3.761	4.242
		High	3.956^a^	0.125	3.707	4.205
Participation	Control	Low	3.513^a^	0.149	3.217	3.808
		High	3.851^a^	0.120	3.613	4.089
	Paper	Low	3.729^a^	0.112	3.507	3.951
		High	4.042^a^	0.131	3.782	4.301
	eCPAT	Low	4.037^a^	0.122	3.795	4.279
		High	3.681^a^	0.126	3.431	3.931
Motivation	Control	Low	4.097^a^	0.126	3.848	4.346
		High	4.185^a^	0.101	3.984	4.386
	Paper	Low	3.968^a^	0.094	3.780	4.155
		High	4.273^a^	0.110	4.054	4.492
	eCPAT	Low	4.274^a^	0.103	4.070	4.479
		High	4.027^a^	0.106	3.816	4.238
Critical Awareness	Control	Low	3.498^a^	0.213	3.076	3.920
		High	3.409^a^	0.172	3.069	3.750
	Paper	Low	3.701^a^	0.160	3.383	4.018
		High	3.838^a^	0.187	3.468	4.209
	eCPAT	Low	3.855^a^	0.174	3.510	4.201
		High	3.382^a^	0.180	3.025	3.739

a *Covariates appearing in the model are evaluated at the following values: MEAN(Pre Self-Efficacy) = 3.9055, MEAN(Pre Intention) = 4.0486, MEAN(Pre Participation) = 3.6875, MEAN(Pre Motivation) = 4.0764, Pre Critical Awareness = 3.4667. No significant main effects for the interaction model were found for youth empowerment (Pillai's Trace = 0.15, F_(10, 194)_ = 11.605, p = 0.107)*.

**Table 7:** Youth advocacy by study condition and technology use.

**Dependent variable youth advocacy**	**Study condition**	**Technology use**	**Mean**	**Std. error**	**95% Confidence interval**	
					**Lower bound**	**Upper bound**
Assertiveness	Control	Low	4.023^b^	0.129	3.768	4.279
		High	3.824^b^	0.111	3.602	4.045
	Paper	Low	3.863^b^	0.097	3.671	4.056
		High	4.200^b^	0.110	3.981	4.418
	eCPAT	Low	3.734^b^	0.117	3.503	3.966
		High	3.709^b^	0.114	3.482	3.935
Perceived sociopolitical control	Control	Low	3.534^b^	0.129	3.278	3.790
		High	3.493^b^	0.112	3.272	3.715
	Paper	Low	3.571^b^	0.097	3.378	3.764
		High	3.802^b^	0.110	3.582	4.021
	eCPAT	Low	3.657^b^	0.117	3.425	3.890
		High	3.434^b^	0.115	3.206	3.661
Advocacy activity	Control	Low	1.959^b^	0.163	1.635	2.283
		High	2.061^b^	0.141	1.781	2.342
	Paper	Low	2.108^b^	0.123	1.864	2.353
		High	2.271^b^	0.140	1.994	2.549
	eCPAT	Low	2.063^b^	0.148	1.770	2.357
		High	1.932^b^	0.145	1.645	2.219
Knowledge	Control	Low	3.890^b^	0.177	3.539	4.241
		High	3.775^b^	0.153	3.471	4.079
	Paper	Low	3.913^b^	0.133	3.648	4.177
		High	4.078^b^	0.151	3.778	4.378
	eCPAT	Low	4.023^b^	0.160	3.705	4.341
		High	3.699^b^	0.157	3.388	4.011

b *Covariates appearing in the model are evaluated at the following values: MEAN(Pre Assertiveness) = 3.8951, MEAN(Pre Perceived Sociopolitical Control) = 3.6111, MEAN(Pre Advocacy Activity) = 2.0046, Pre Knowledge = 3.7407. No significant main effects for the interaction model were found for youth advocacy variables (Pillai's Trace = 0.11, F_(8, 192)_ = 1.449, p = 0.179)*.

The final research question explored youth impressions of and preferences for paper vs. mobile technology tool formats. Youth impressions of the Paper and eCPAT tools were not statistically different (*t*_(86)_ = 0.397, *p* = 0.69). To further understand youth preferences for paper vs. mobile technology tools, we analyzed data from the delayed intervention (Both) group that tested both formats (*n* = 31). As shown in Table 8, the majority of youth who tested both conditions thought that the eCPAT app was easier to use (71.0%), enjoyed using the eCPAT app the most (80.6%), liked the eCPAT app format the best (77.4%), and would prefer to use the eCPAT app in future projects (80.6%). In addition, 93.5% of youth indicated they would use the eCPAT application in future projects.

**Table 8:** Youth tool format preferences.

**Preference Item**	**Paper CPAT**	**eCPAT app**	**I liked both equally**	**I don't like either**
Which format was easier to use?	9.7%	71.0%	16.9%	3.2%
Which format did you enjoy using the most?	6.5%	80.6%	9.7%	3.2%
Which format would you want to use in future projects?	3.2%	80.6%	12.9%	3.2%
Overall, which format did you like the best?	9.7%	77.4%	12.9%	0.0%

## Discussion

With the dramatic increase in childhood obesity rates over the last three decades, it is important to explore population-level solutions to youth physical inactivity (3, 66). Modifying the built environment of neighborhoods and communities is recognized as a promising solution (13, 14). However, civically engaging and empowering community members, especially youth, in healthy PSE change initiatives is essential to successful efforts (16, 67). Recent youth community health PAR paradigms have incorporated technology as a way to engage and empower youth to make healthy changes in their communities (26, 27). Electronic data collection provides numerous youth benefits within PAR frameworks (Table 1) as well as improved data integrity via electronic data collection (i.e., reducing data loss, response validation). The current study extends the literature by exploring the effects of youth using a mobile technology data collection tool with respect to their reported levels of usability, empowerment, advocacy, and preference.

Baseline levels of youth access to technology revealed that the majority of study youth had access to multiple types of technology, especially mobile technology such as smartphones, tablets, or iPads. This finding is similar to a recent national survey showing high percentages of youth access to smartphones (47%), tablets (23%), or laptops (90%), as well as growing use of mobile technology applications (58%) and social networking sites (81%) (68). While mobile technologies are pervasive in our society, especially among youth, few apps are used to engage youth in health policy or environmental change. Our study found that youth were willing to utilize mobile technology for healthy PAR activities such as communication and advocacy efforts. This finding substantiates previous inferences that mobile technology is indeed a viable platform to civically engage youth in community health advocacy and promotion efforts. Likewise, these findings confirm the need to further develop and research the effects of such mobile applications and other technology tools for engaging youth in PSE change (28).

Overall, youth indicated above average usability for both data collection tool formats used in this study (i.e., paper CPAT and eCPAT mobile application). This confirms that original efforts to create a user-friendly community park audit tool (CPAT) for use among diverse community members were efficacious (45). Exploring the effectiveness of using mobile technology vs. paper-pencil methods on indicators of youth empowerment or advocacy, we did not find significant differences between the eCPAT, Paper, or Control groups post project, controlling for pre-project scores. This result is inconsistent with previous research that has shown numerous benefits of using technology within youth PAR frameworks (24, 26, 37, 39, 40). Although our results illustrated that youth in the eCPAT group exhibited positive changes for six out of nine youth empowerment and advocacy variables, our study may have been underpowered to detect significant differences (64). Moreover, this pilot project only involved youth *collecting* observational park audit data. While all youth were able to successfully submit data upon audit completion, at the time of post evaluation, youth had not discussed, shared, or acted upon any of the data they had collected. Even though utilization of the eCPAT application for data collection purposes potentially fulfills multiple characteristics of successful youth PAR (e.g., engages and challenges youth, increases critical awareness of community issues), it may be that for youth to experience increases in levels of empowerment or advocacy, additional elements of youth PAR must be accomplished before “meaningful participation” is achieved (20, 24). Therefore, future research will seek to integrate eCPAT mobile technology use into broader action-oriented projects that leverage benefits of technology, such as improved adult-youth communications, equitable power sharing, and increased political or social agency (24, 37, 40).

Overall, this study found high levels of regular mobile technology use among youth (i.e., over 80% of the youth sample used mobile technology at least once a week). We found no significant interaction effect between regular mobile technology use and study condition on post-project levels of youth empowerment or advocacy. This result suggests that mobile technology competency may not be an issue in youth populations as compared to what Campbell and colleagues found to be true in adults (52). Nonetheless, future youth projects may need to consider mobile technology competency prior to integrating the eCPAT tool into PAR activities, especially among low income populations who may not have as abundant access or use of such technologies (68). In such instances, a brief introduction to mobile technology and, specifically, eCPAT capabilities may be warranted. Moreover, our study only viewed the technology moderator in terms of understanding how well youth might be able to adapt to using the eCPAT mobile technology tool format. As noted by Farnham and colleagues, it may be that youth's experience using mobile technology for specific purposes in the public/social domain (i.e., blogs, wikis, Twitter) may be more likely to influence the relationship between youth using mobile technology for PAR and resulting levels of youth empowerment or advocacy (51). Consequently, future research with the eCPAT tool should consider ways that youth can publicly share data collection efforts to enhance youth's feelings of community interaction for health advocacy.

Finally, our study found that while the youth had positive to very positive impressions of both the paper-pencil and eCPAT mobile app tools, the vast majority of youth who experienced both tools preferred the eCPAT mobile application. Furthermore, almost all youth indicated that they would use the eCPAT application in future projects. This finding confirms the feasibility of the eCPAT mobile application and provides preliminary evidence that the use of eCPAT mobile technology could facilitate the PAR process among youth populations (46, 53). As youth become more adept with electronic collection and utilization of PAR data, future research should seek to update this study's findings.

### Limitations

This study had several limitations which provide direction for future research. For example, while our pilot study recruited 50 youth per condition, final group totals were lower than desired. Low sample size may have reduced our power to detect changes between groups. Likewise, the use of only one location may have inhibited our ability to generalize findings to youth beyond our study. Future research should consider increasing sample size and geographic locations to address these considerations. The voluntary nature of study participation or the recruitment methods employed could have contributed to bias in attracting youth interested in such a project or topic. However, as mentioned earlier, study participant characteristics were similar to those of youth in Greenville County. Further, randomization of youth into study conditions reduced potential bias on key variables; indeed, analyses of multiple sample characteristics indicated no differences between the three study conditions on the majority of variables. The only difference found between groups was for access to laptops. Youth technology use has increasingly taken the form of newer technologies (e.g., smartphones, tablets) which were more relevant to this study. Likewise, self-report survey measures and monetary incentives for project completion could lend to social desirability bias. However, our measures included multiple items for youth technology use, empowerment or advocacy that have previously shown good validity and reliability (50, 61, 62). Further, use of a no treatment control group pretest/posttest design allowed us to understand naturally occurring changes in key measures and explore potential causal effects of technology on youth empowerment and advocacy. Finally, as noted above, this study only explored the effect of mobile technology in youth PAR in the context of environmental data collection. Future research should explore the use of eCPAT mobile technology with a large number of youth as part of action-oriented community health projects.

## Conclusion

Overall, technology is becoming a staple among teens that cannot be ignored. Rather, researchers should capitalize on the proliferation of mobile devices to meet youth on digital platforms where they are spending their time. A growing body of research indicates that technology supports essential dimensions of youth PAR empowerment models while combating common PAR issues such as apathy, lack of trust, and power-sharing (24, 34, 35). While the present study did not show significant effects or interaction of technology use between study conditions, our results illustrated that youth exhibited positive changes in youth empowerment and advocacy variables pre to post project. Moreover, youth indicated high levels of eCPAT tool usability and a strong preference for using mobile devices within youth PAR frameworks. In summary, eCPAT mobile technology should be viewed as a potential strategy for increasing youth engagement and empowerment in PAR for health promotion (27, 28). Future dissemination of this research will integrate the eCPAT application as a critical component of the Healthy Young People Empowerment (HYPE) Project, (46) a broader youth-led, community-based participatory research project to improve youth and community health. Given the ubiquity of smartphones and other electronic devices among both adolescents and adults, (68) the eCPAT application also has potential to be distributed and used widely by both the general public and professionals alike to achieve successful community engagement in healthy PSE change efforts.

## Author contributions

GB with the help of AK conceptualized and implemented all aspects of the study. GB drafted the manuscript under the supervision of AK. BS led mobile technology development and contributed to data management and manuscript revisions. SWS, SW, and GT-M contributed to study planning, data analysis and interpretation, and manuscript revisions.

### Conflict of interest statement

The authors declare that the research was conducted in the absence of any commercial or financial relationships that could be construed as a potential conflict of interest.
